# Osteocalcin binds to a GPRC6A Venus fly trap allosteric site to positively modulate GPRC6A signaling

**DOI:** 10.1096/fba.2024-00025

**Published:** 2024-08-14

**Authors:** Rupesh Agarwal, Ruisong Ye, Micholas Dean Smith, Jeremy C. Smith, L. Darryl Quarles, Min Pi

**Affiliations:** ^1^ Oak Ridge National Laboratory Center for Molecular Biophysics University of Tennessee Oak Ridge Tennessee USA; ^2^ Department of Biochemistry and Cellular and Molecular Biology University of Tennessee Knoxville Tennessee USA; ^3^ Department of Medicine University of Tennessee Health Science Center Memphis Tennessee USA

**Keywords:** extracellular domain, G protein‐coupled receptor (GPCR), GPRC6A, homology modeling, isoform‐selectivity, mutagenesis, negative allosteric modulator, osteocalcin, positively allosteric modulator

## Abstract

GPRC6A, a member of the Family C G‐protein coupled receptors, regulates energy metabolism and sex hormone production and is activated by diverse ligands, including cations, L‐amino acids, the osteocalcin (Ocn) peptide and the steroid hormone testosterone. We sought a structural framework for the ability of multiple distinct classes of ligands to active GPRC6A. We created a structural model of GPRC6A using Alphafold2. Using this model we explored a putative orthosteric ligand binding site in the bilobed Venus fly trap (VFT) domain of GPRC6A and two positive allosteric modulator (PAM) sites, one in the VFT and the other in the 7 transmembrane (7TM) domain. We provide evidence that Ocn peptides act as a PAM for GPRC6A by binding to a site in the VFT that is distinct from the orthosteric site for calcium and L‐amino acids. In agreement with this prediction, alternatively spliced GPRC6A isoforms 2 and 3, which lack regions of the VFT, and mutations in the computationally predicted Ocn binding site, K352E and H355P, prevent Ocn activation of GPRC6A. These observations explain how dissimilar ligands activate GPRC6A and set the stage to develop novel molecules to activate and inhibit this previously poorly understood receptor.

## Graphical abstract

Basis for multiple ligands activation of GPRC6A derived from predicted 3D structures of GPRC6A. The model proposes an orthosteric ligand binding site in the bilobed Venus fly trap (VFT) domain and two allosteric sites, one in the VFT and the other in the 7 transmembrane (7TM) domain. Osteocalcin peptides act as a positive allosteric modulator (PAM) for GPRC6A by binding to a site in the VFT that is distinct from the orthosteric site for calcium and L‐amino acids.

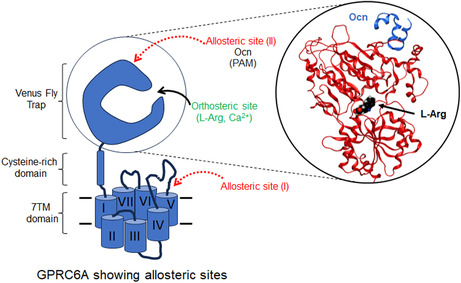

**Abbreviations**
CaSR: calcium‐sensing receptor
cAMP: cyclic adenosine monophosphate
CRD: cysteine rich domain
ERK: extracellular‐signal‐regulated kinase
GPRC6A: G protein‐coupled receptor family C group 6 member A
mGluR: metabotropic glutamate receptor
NAM: negative allosteric modulator
Ocn: osteocalcin
PAM: positive allosteric modulator
VFT: venus flytrap; TM: transmembrane

## 1 INTRODUCTION

Osteocalcin (Ocn) is a 49‐residue protein in humans that is mainly produced by osteoblasts in bone. A vitamin K‐dependent carboxylated form of Ocn comprises much of the non‐collagenous bone protein matrix, while an undercarboxylated peptide (unOcn) is released into the circulation to function as a putative hormone. unOcn is proposed to regulate energy metabolism through direct effects on metabolic functions in multiple target tissues and by indirect effects mediated by an ensemble of metabolically active hormones whose release is stimulated by unOcn.^1^, ^2^, ^3^, ^4^, ^5^ Indeed, unOcn is reported to stimulate insulin secretion and β‐cell proliferation in the pancreas,^5^ fibroblast growth factor 21 (FGF‐21) release and regulation of glucose and fat metabolism in liver hepatocytes,^6^ stimulate interleukin 6 (IL‐6) secretion and glucose utilization in skeletal muscle myocytes,^7^ as well as induce adiponectin release and lipolytic activity of adipocytes in white fat,^8^ testosterone production from testicular Leydig cells,^2^, ^6^ and glucagon‐like peptide 1 (GLP‐1) secretion from gastrointestinal enterocytes.^9^, ^10^

The metabolic effects of Ocn are proposed to be mediated through activation of GPRC6A, a member of the Family C G‐protein coupled receptors.^5^, ^11^, ^12^ The respective phenotypes in mouse models of loss‐ and gain‐of Ocn and GPRC6A function are similar.^2^, ^3^, ^4^ GPRC6A is activated by multiple ligands. Basic L‐amino acids (such as L‐Lys, L‐Arg, and L‐ornithine) and divalent cations (such as Ca^2+^ and Mg^2+^) are likely bind to a putative orthosteric binding site in the extracellular domain, while positive allosteric modulators (PAMs) of GPRC6A include testosterone, NPS‐568, and di‐phenyl and tri‐phenyl compounds and may bind to the 7TM.^13^, ^14^ Gallate and epigallocatechin 3‐gallate (ECGC), which are natural products in green tea, respectively activate and inhibit GPRC6A.^15^

The structural basis for GPRC6A interaction with multiple ligands is incompletely understood. Family C GPCRs are characterized by their similarity in 7 transmembrane (7TM) domain sequences, a necessity for dimerization to function, and in most cases, the existence of a large N‐terminal ligand‐binding Venus flytrap (VFT) domain.^16^ Recently, high resolution structural data of the mGluR5^17^ and CaSR,^18^, ^19^, ^20^, ^21^ which are related to GPRC6A, have identified how various ligands and drug molecules target the VFT and 7TM. For example, mGluR5 has an orthosteric binding site for L‐glutamate in the VFT, and two positive modulating allosteric (PAM) sites, one located in the VFT and the other in the 7TM.^17^ The allosteric site located in the VFT was discovered by a nanobody, Nb43, that binds to Helix L and the L‐M loop of mGluR5 to potentiate orthosteric agonist binding and receptor activation. Similarly, elucidation of the 3D structure of CaSR identified in the VFT an orthosteric Ca^2+^ ion binding site that acts as a composite agonist with L‐amino acids, such as L‐tryptophan, to stabilize the closure of the active VFT.^18^ CaSR also has an allosteric site at the dimeric interface of the VFT (proximal to residue D248) to which the peptide etecalcitide binds and acts as a positive allosteric modulator (PAM). In CaSR, additional small molecule drugs act as PAMs (e.g., calcimimetics), or negative allosteric modulators (NAMs) (e.g., calcilytic) via a second PAM site in the 7TM.^18^, ^19^, ^20^, ^21^

Based on this new structural information of these closely related receptors, we developed and tested a homology model for the activation of GPRC6A by orthosteric and allosteric ligands. We also developed a structural model for Ocn activation of GPRC6A and confirmed Ocn activation sites by studying isoforms with deleted segments of the extracellular domain, mapping the peptide fragments of Ocn necessary for activation of GPRC6A, and examining if Ocn peptides bind to the VFT to modulate GPRC6A function.

## 2 MATERIALS AND METHODS

### 2.1 Homology model construction and docking details

GPRC6A and Ocn structures were modeled using AlphaFold2,^22^, ^23^, ^24^ which is an AI system developed by DeepMind to predict a 3D structure of a protein from its amino acid sequence. Multiple sequence alignment (MSA) was performed using the Jackhammer method. The “max_recycles” parameter, *r* which controls the maximum number of times the structure is fed back into the neural network for refinement, was set to 3. All the other parameters were set to default. The final models were ranked using the pLDDT score. Glycans were not added as the details are not well known. For docking, HADDOCK 2.4^25^ was used to dock the GPRC6A‐VFT to Ocn. GPRC6A residue numbers 334, 335, 336, 337 and mature Ocn residue numbers 21, 22, 23, 24, 25, 26, and 27 were used to guide the docking. The best 4 models from the top cluster (containing 133 models) with HADDOCK score of −81.2 ± 1.1 and *Z*‐score of −1.5 were analyzed.

### 2.2 Measurement of total and phospho‐ERK by ERK elisa analysis

Ocn was purified from bovine tibial bone extracts.^26^, ^27^ Decarboxylated Ocn was produced by treating Ocn in vacuo at 110°C.^27^, ^28^, ^29^ The purity and decarboxylation state were confirmed by native gel electrophoresis,^26^ or by blotting followed by reaction with 4‐diazobenzene sulfonic acid staining for γ‐carboxyglutamic acid.^27^, ^28^ The human Ocn fragments including full human Ocn (Ocn 1–49; 49 aa), Ocn 8–43 (36 aa), Ocn 1–7 (7 aa), Ocn 8–19 (12 aa), Ocn 20–29 (10 aa), Ocn 30–39 (10 aa) and Ocn 44–49 (6 aa) were synthesized by BioMatik USA (Wilmington, DE, USA).

All culture reagents were from Invitrogen (Waltham, MA, USA). Human embryonic kidney HEK‐293 cells were obtained from American Type Culture Collection and cultured in DMEM medium supplemented with 10% fetal bovine serum and 1% Penicillin/Streptomycin (P/S). Briefly, HEK‐293 cells transfected with/without human GPRC6A isoforms cDNA plasmids were starved by overnight incubation in serum‐free DMEM/F12 containing 0.1% bovine serum albumin (BSA) and stimulated with various ligands at different doses. ERK activation was assessed 20 min after treatment by using ERK1/2 (phospho‐T203/Y204) ELISA Kit (Invitrogen; Waltham, MA, USA) corrected for the amount of total ERK using ERK1/2 (Total) ELISA Kit (Invitrogen; Waltham, MA, USA) to measure ERK levels.

### 2.3 Measurement of cAMP accumulation

HEK‐293 transfected with human GPRC6A isoform cDNA plasmids cells (10^5^ cells/well)^30^ were cultured in 24‐well plates in DMEM supplemented with 10% fetal bovine serum and 1% penicillin/streptomycin (100 U/mL of penicillin and 100 μg/mL of streptomycin) for 48 h followed by 4 h incubation in DMEM/F12 containing 0.1% BSA and 0.5 mM IBMX to achieve quiescence and preventing cAMP degradation. Quiescent cells were treated with vehicle control, various ligands at concentration as indicated for 40 min at 37°C. Then, the reaction was stopped, and the cells lysed with 0.5 mL 0.1 N HCl. cAMP levels were measured by using Cyclic AMP EIA kit (Cayman Chemical; Ann Arbor, MA, USA) following the manufacture's protocol.

### 2.4 Mouse and serum biochemistry

8‐week‐old wild‐type (*Gprc6a* ^*+/+*^) and global knock out (*Gprc6a* ^*−/−*^) mice were injected intraperitoneally with Ocn 20–29 (2 nmol/g body weight), or vehicle (saline; 10 μL/g body weight). Serum was collected at 24 h after intraperitoneal injection. Blood glucose levels were measured by using blood glucose strips and the Accu‐Check glucometer as described.^7^, ^31^ A mouse IL‐6 ELISA Kit was purchased from Invitrogen (Waltham, MA, USA). Rat/Mouse FGF‐21ELISA Kit was obtained from Millipore Sigma (Burlington, MA, USA).

The *Gprc6a* ^*−/−*^ mouse model was created by replacing exon 2 of the GPRC6A gene with the hygromycin resistance gene, as described previously.^32^ Mice were maintained and used in accordance with recommendations as described (National Research Council 1985; Guide for the Care and Use of Laboratory Animals Department of Health and Human Services Publication NIH 86–23, Institute on Laboratory Animal Resources, Rockville, MD) and following guidelines established by the University of Tennessee Health Science Center Institutional Animal Care and Use Committee. The animal study protocol was approved by the institutional review boards at University of Tennessee Health Science Center Institutional Animal Care and Use Committee.

### 2.5 Site‐directed mutagenesis

In‐vitro mutagenesis by PCR‐mediated recombination QuikChange II XL Site‐Directed Mutagenesis Kit (Agilent) was performed using the cDNA of human GPRC6A cloned in the vector pcDNA3 (Invitrogen) as a template. The primer sets are as follows. K352E.sen: GTGACAGTCACGAACTCTTACATG; K352E.antisen: CATGTAAGAGTTCGAGACTGTCAC; H355P.sen: CAAACTCTTACCTGAATATGCCATG; H355P.antisen: CATGGCATATTCAGGTAAGAGTTTG; K352E/H355P.sen: GTGACAGTCACGAACTCTTACGTGAATATGC; and K352E/H355P.antisen: GCATATTCAGGTAAGAGTTCGTGACTGTCAC. All mutants were confirmed by sequencing.

### 2.6 Statistics

We evaluated differences between groups with the Student's *t* test, and for multiple groups by two‐way ANOVA, followed by *a post‐hoc* Tukey's test. Significance was set at *p* < 0.05. All values are expressed as means ± SEM. All computations were performed using the Statgraphic statistical graphics system (STSC Inc., Rockville, MD, USA).

## 3 RESULTS

### 3.1 Structural modeling of GPRC6A isoforms and L‐Arginine orthosteric ligand binding

**FIGURE 1:**
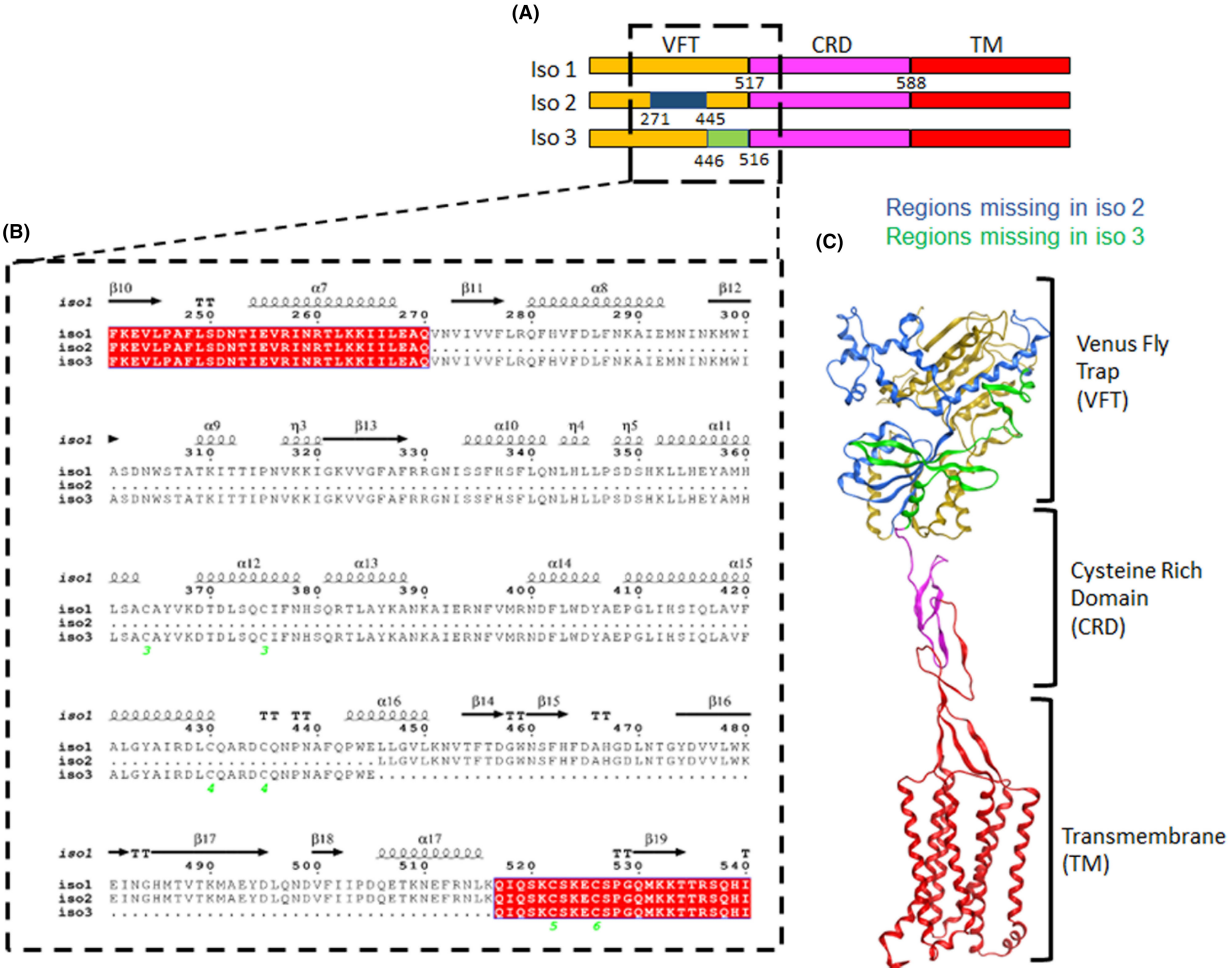
Sequence and structure of GPRC6A (A) The GPRC6A isoform 1, 2, and 3 sequences showing that CRD (in magenta) and TM regions (in red) are identical in the three isoforms and the missing regions (in blue and green for isoform 2 and 3 respectively). are in VFT (in yellow) for Isoform 2 and 3; (B) sequence alignment of Venus flytrap (VFT) domain showing missing regions of isoform 2 and 3; (C) structure model of GPRC6A showing the three domains and the missing region of isoform 2 and 3.

GPRC6A exists as three isoforms: isoform 1 represents the full‐length receptor, and two alternatively spliced receptor isoforms 2 and 3 are characterized by respective deletions of exon 3 and exon 4 segments that encode regions of the VFT domain^33^ (Figure 1A). To identify regions that differ between the isoforms, we aligned the protein sequences of isoforms 1, 2 and 3 of GPRC6A (Figure 1B; Figure S1). The TM domains of these three isoforms have 100% sequence identity, but isoforms 2 and 3 are missing residues 271–445 and 446–516 in the extracellular domain, respectively. These naturally occurring differences in the VFT region were used in functional assays to probe ligand binding domains in GPRC6A (vide infra).

To derive the structure of GPRC6A, we used AlphaFold2^22^, ^23^, ^24^, ^34^ to model the full‐length isoform 1 (Figure 1C). AlphaFold2 is an AI‐based technique that is close‐to‐experimental accuracy in protein structure prediction. These calculations confirmed that GPRC6A has 3 domains: TM, CRD and VFT, similar to the related mGluR5 and CaSR Cryo‐EM structures. The residues deleted in the isoforms 2 and 3 correspond to different segments of the VFT domain. In the case of isoform 2, a large segment of the VFT domain structure (~ 174 residues) is deleted, whereas a relatively small region (~ 70 residues) is missing in isoform 3 (Figure S1).

In GPRC6A isoform 1, we identified a probable orthosteric amino acid ligand site near residues Tyr148, Ser149, Thr172, Asp303 in the VFT based on structure superimposition with mGluR5 and CaSR (Figure S2A,B). In contrast, in isoform 2, the predicted orthosteric ligand binding site based on aligning orthosteric bound structures of mGluR5 and CaSR structures is missing, foretelling a loss‐of‐function to the orthosteric ligand, L‐Arg (vide infra) (Figure S2C). The predicted orthosteric ligand binding site is retained in isoform 3, and hence this isoform is predicted to be responsive to orthosteric ligands (vide infra).

**FIGURE 2:**
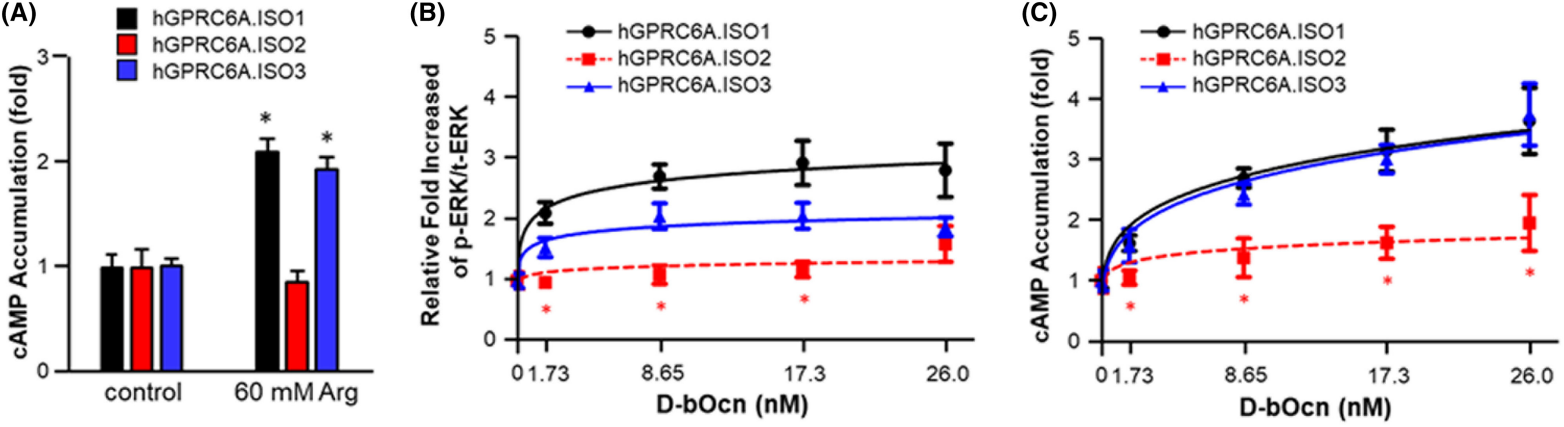
Activities of GPRC6A isoforms on response to Ocn. (A) Comparison of effects of L‐Arg on GPRC6A isoforms mediated cAMP accumulation. Comparison of dose‐dependent effects of Ocn on GPRC6A isoforms mediated ERK activation (B) and cAMP accumulation (C) HEK‐293 cells were transfected with cDNA plasmids of GPRC6A isoform 1, 2 or 3 for 48 h, after incubated in Dulbecco's modified Eagle's medium/F‐12 containing 0.1% bovine serum albumin quiescence media for 4 h, then exposed to L‐Arg or Ocn at indicated concentrations for 15 min for ERK activation, or 40 min for cAMP accumulation details as described under “Methods.” * indicates a significant difference from control and stimulation groups at *p* < 0.05. In (A), the control is data from not L‐Arg stimulation for each isoform. In (B, C), the control is data from not Ocn stimulation for each isoform.

To test these predictions, GPRC6A isoforms 1, 2, or 3 were transfected into HEK‐293 cells and similar levels of protein membrane expression were confirmed by Western blot analysis (Figure S3A). Receptor activation was measured by cAMP accumulation in response to the orthosteric ligand L‐Arg in the presence of 1 mM calcium. Indeed, isoform 2 resulted in a loss of L‐Arg stimulation of cAMP, whereas isoform 3 retained responsiveness to L‐Arg activation, similar to isoform 1 (Figure 2A).

### 3.2 GPRC6A isoforms 2 and 3 have attenuated responses to full‐length Ocn (1–49)

The location of the Ocn binding site in GPRC6A is still not well understood. To explore the role of the VFT in mediating the response to Ocn, we compared the ability of Ocn at concentration of 1.73 nM (10 ng/mL), 8.65 nM (50 ng/mL), 17.3 nM (100 ng/mL) and 26.0 nM (150 ng/mL) to activate the three GPRC6A isoforms (Figure 2B,C). Ocn in the presence of 1 mM calcium activated GPRC6A isoform 1 in a dose dependent manner, consistent with previous reports.^5^ GPRC6A isoform 2 exhibited an attenuated ERK response and a complete loss of cAMP activation in response to Ocn treatment. In contrast, GPRC6A isoform 3 also showed attenuation of Ocn‐induced ERK stimulation but maintained the cAMP response to Ocn (Figure 2B,C). Taken together, loss‐of Ocn responses in isoforms 2 suggest that this region of the VFT is necessary for the full‐length Ocn peptide to activate GPRC6A.

### 3.3 Evidence that the Ocn 20–29 fragment is sufficient to activate GPRC6A

**FIGURE 3:**
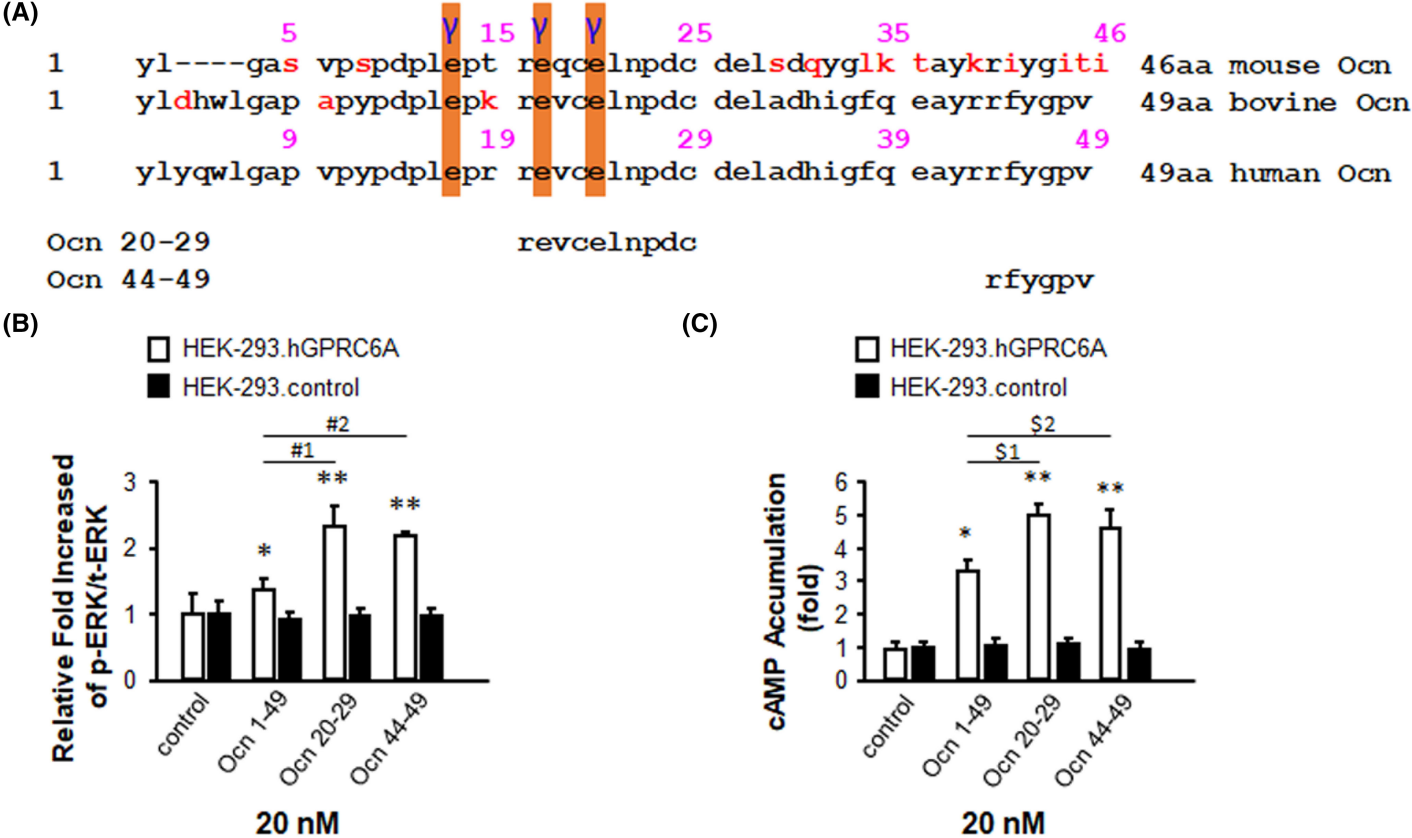
The activities of human Ocn peptide fragments. (A) The sequence alignment of full length mouse, bovine and human Ocn (upper panel), and synthesized peptide fragments of human Ocn (bottom panel). Red alphabets indicate different amino acids compared to human Ocn sequence. “γ” indicates carboxylation sites in Ocn. Comparison of the activities of synthesized fragments of human Ocn (at 20 nM) by ERK phosphorylation (B) and cAMP accumulation (C) in HEK‐293 transfected with vector pcDNA3 (black bar) and HEK‐293 cells transfected with pcDNA3‐hGPRC6A cDNA (white bar). The ERK phosphorylation was measured 15 min and cAMP accumulation was measured 40 min for Ocn fragments at concentration as indicated at indicated stimulation in HEK‐293 cells transfected with pcDNA3‐hGPRC6A cDNA after 4 h quiescence. * and ** indicate a significant difference from control (HEK‐293.hGPRC6A cells stimulated buffer only, did not with any Ocn peptides) and Ocn peptides stimulation groups at *p* < 0.05 and *p* < 0.01, respectively. #1 indicate a significant difference from peptide Ocn 1–49 and peptide Ocn 20–29 groups at *p* < 0.01. #2 indicate a significant difference from peptide Ocn 1–49 and peptide Ocn 44–49 groups at *p* < 0.01. $1 indicate a significant difference from peptide Ocn 1–49 and peptide Ocn 20–29 groups at *p* < 0.05. $2 indicate a significant difference from peptide Ocn 1–49 and peptide Ocn 44–49 groups at *p* < 0.05.

To map the regions of Ocn necessary for bioactivity, we synthesized Ocn fragments. Ocn fragments (Figure 3A)^11^, ^25^ for their ability to activate the full length GPRC6A isoform 1 (Figure 3B,C) transfected into HEK293 cells. Ocn peptide activation was measured by assessing both ERK (Figure 3B) and cAMP (Figure 3C) signaling. We found that the decapeptide fragment of Ocn 20–29 (REVCELNPDC) located in the middle of the protein and the C‐terminal hexapeptide Ocn 44–49 (RFYGPV) exhibited high activity (Figure 3B,C; Figure S5A).

**FIGURE 4:**
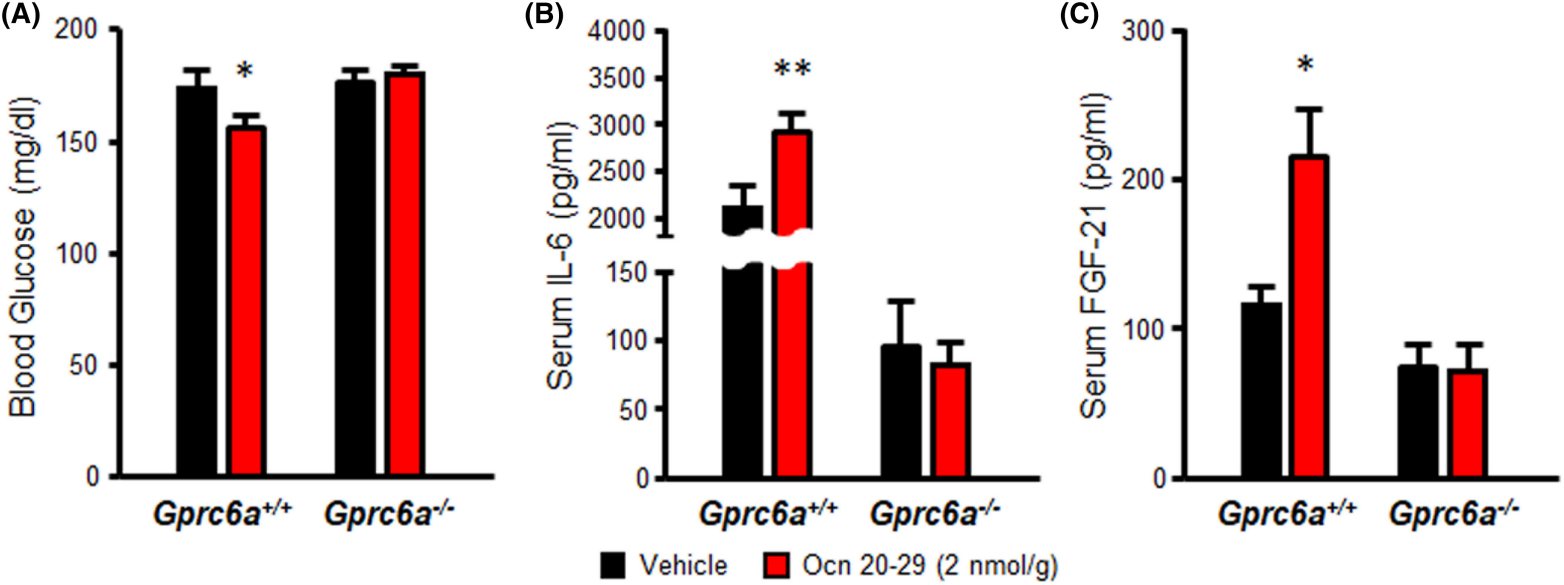
The effects of Ocn fragment, Ocn 20–29 on GPRC6A‐mediated regulation of blood glucose, serum IL‐6 and FGF‐21 levels in mouse. The biological activities of Ocn 20–29 were assessed 24 h after intraperitoneal injection of 2 nmol/g the Ocn 20–29 peptide (red bar) or vehicle (saline, black bar) in 8 week‐old *Gprc6a* ^*+/+*^ or *Gprc6a* ^*−/−*^ mice by measuring blood glucose (A), serum IL‐6 (B), and FGF‐21 (C) Ocn 20–29 significantly decreased the blood glucose level, and increased serum IL‐6 and FGF‐21 levels compared to vehicle in wild‐type mice. These responses were lost in *Gprc6a* ^*−/−*^ mice. Superscripts * and ** indicate a significant difference from vehicle and Ocn 20–29 injection groups (*n* ≥ 5) at *p* < 0.05 and *p* < 0.01; Vehicle group vs. Ocn 20–29 treatment group, respectively.

To gain preliminary insights into the bioactivity of Ocn 20–29 fragment in vivo, we injected the synthetic Ocn 20–29 peptide (2 nmol/g) into wild type *Gprc6a* ^*+/+*^ and *Gprc6a* ^*−/−*^ mice by the intraperitoneal route,^31^ and collected blood and serum at 24 h. We found that Ocn 20–29 at this dose reduced blood glucose levels in wild‐type mice by ~10% at 24 h, whereas the vehicle (saline) had no effect on blood glucose (Figure 4A). Consistent with prior reports of Ocn effects to stimulate IL‐6 release from skeletal muscle^7^ and to increase FGF‐21 circulating levels in mice,^11^ Ocn 20–29 at a dose of 2 nmol/g IP also resulted in a significant increase in serum IL‐6 and FGF‐21 levels in wild type *Gprc6a* ^*+/+*^ mice, but not in *Gprc6a* knockout (*Gprc6a* ^*−/−*^) mice (Figure 4B,C). While more comprehensive dose and time‐dependent effects are required to fully characterize the effects of Ocn peptides on hormone secretion and glucose, fat, and amino acid metabolism, these positive in vivo studies support our models of Ocn activation of GPRC6A.

### 3.4 Protein–protein docking to generate GPRC6A‐VFT: Ocn complex structure

As noted above, there are two PAM sites in the related receptors mGluR5 and CaSR, one in the 7TM domain targeted by small molecules and another in the VFT targeted by the protein nanobody^17^ and the etecalcitide peptide.^21^ Specifically, the PAM function of Nb43 is mediated by a nanobody binding site near a helix in the VFT domain of the mGluR5 as defined by Cryo‐EM.^17^ Aligning the structures of the allosteric ligand bound VFTs mGluR5 (PDB 6N4Y) and CaSR (PDB 7E6U, 7M3G) with our model of GPRC6A and its isoforms indicated that the dimeric interface found to bind etecalcitide and NAM in CaSR was invariant across the GPRC6A isoforms, while the binding pocket homologous to the mGluR5 nanobody PAM site was absent in GPRC6A's isoform 2 (Figure S6A). We hypothesized that, given the loss of Ocn associated function in isoform 2, the pocket homologous to the mGluR5 binding site may serve as the binding site for Ocn in GPRC6A. Using this site as our guide, we used HADDOCK 2.4^25^ to generate a model of the GPRC6A‐VFT: Ocn complex. The top 4 scoring structures within the calculated top cluster were then subjected to additional analysis and visual inspection.

**FIGURE 5:**
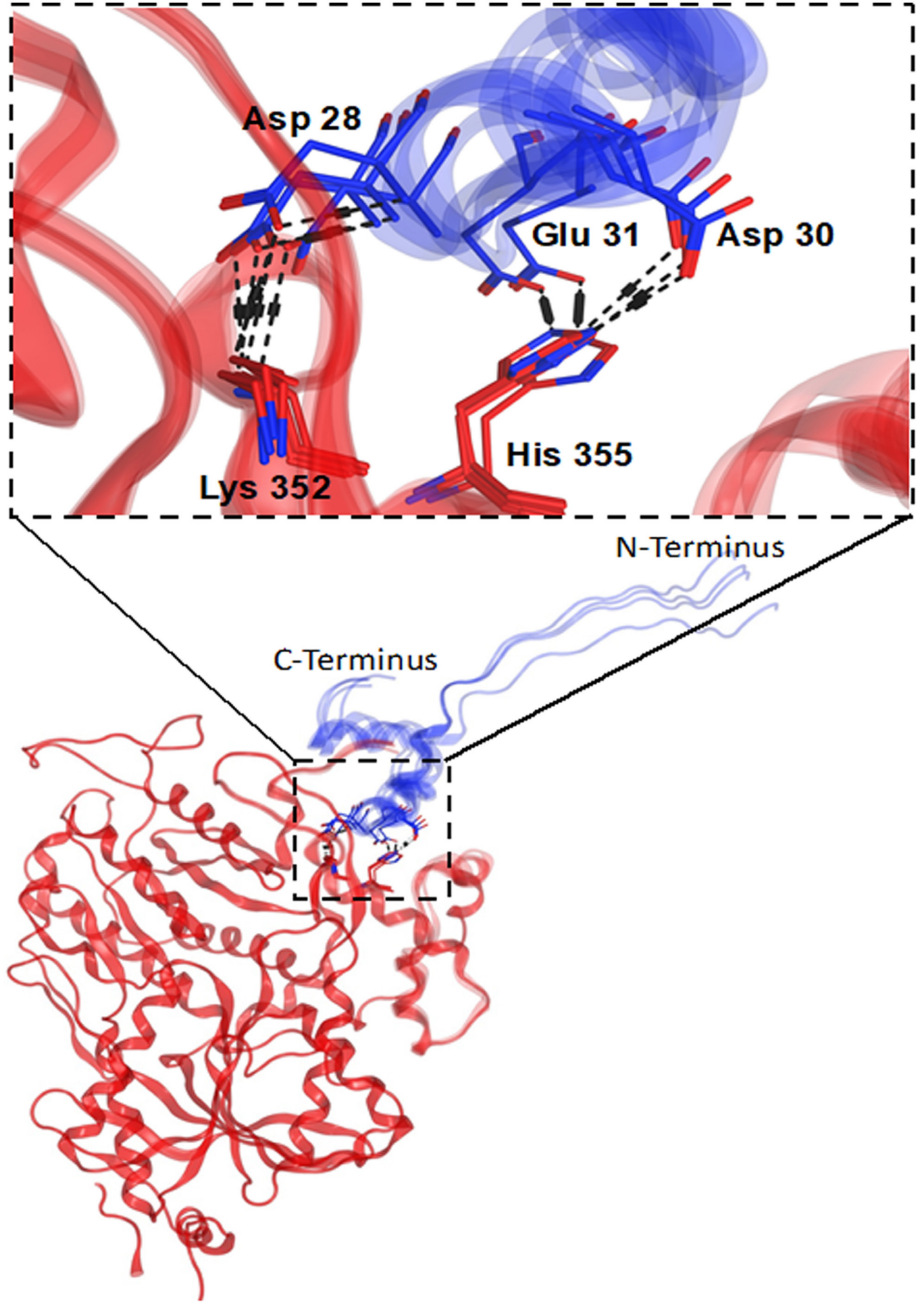
Superimposition of the top 4 models of GPRC6A's VFT domain (in red) and Ocn (in blue) complex generated from HADDOCK. The key residue interactions present in the common in helix region (352–363) of GPRC6A's VFT and residues 20–29 of Ocn.

Our analysis revealed that the GPRC6A‐VFT forms multiple hydrogen bonds (a list is provided in Table 1) with Ocn in all the models. Residues 20–29 from the highest active Ocn fragment also form multiple hydrogen bonds with GPRC6A residues (Gln19, Asp26, Gln381, Lys352) (Figure 5) and is also in proximity (within 4 Å) to other residues (Ala385, Arg382, Asp26, Asp349, Gln19, Gln22, Gln61, Glu356, Glu62, His351, His355, Pro20, Ser348, Ser350, Val28), which can form other potential electrostatic interactions. Moreover, the Ocn model shows that there is a disulfide bond between residues Cys23 and Cys29 (Figure S6B), both of which are present in the Ocn fragment. This is predicted to provide structural integrity to the fragment and would potentially fold similarly to the full length Ocn peptide. In analyzing the interactions between the GPRC6A‐VFT and Ocn, we observe that two GPRC6A‐VFT residues, Lys352 and His335, in the helix region (352–363) are common in all the models (Figure 5; Table 1). These residues form hydrogen bonds with Ocn residues Asp28, Asp30 and Glu31 and are near several other residues present in 20–29 region of Ocn that was the most active fragment.

**TABLE 1:** List of hydrogen bonds between GPRC6A‐VFT and Ocn in best 4 structures from the top cluster from HADDOCK.

GPRC6A residue id	Ocn residue id	Distance (Å)	Model number
Asn389	Asp34	2.79	1
Asp26	Tyr46	2.73	1
His355	Asp30	2.93	1
Lys352	Asp28	3.31	1
Ser348	Glu31	2.67	1
Asp26	Asn26	2.81	2
Asp26	Phe45	3.83	2
Asp26	Tyr46	2.73	2
Gln381	Glu24	2.8	2
Gln381	Pro27	3.06	2
His355	Asp30	3.38	2
His355	Glu31	2.8	2
Lys352	Asp28	3.12	2
Ser348	Glu31	2.71	2
Ala385	Asp30	3.47	3
Asn389	Asp34	2.81	3
Asn389	His35	4.7	3
Asp26	Asn26	2.81	3
Gln19	Pro18	3.89	3
Gln19	Glu21	3.03	3
Gln381	Glu24	2.81	3
His355	Asp30	2.91	3
Lys352	Asp28	3.18	3
Pro20	Pro18	3.48	3
Ser348	Glu31	3.6	3
Ala385	Asp30	3.57	4
Asp26	Phe45	3.38	4
Gln19	Pro18	3.86	4
Gln381	Glu24	2.8	4
Gln381	Asp30	2.87	4
His355	Glu31	2.75	4
Lys352	Asp28	3.06	4
Pro20	Pro18	3.06	4
Ser348	Glu31	2.71	4

### 3.5 Validation of the complex structure using single point mutagenesis

To test the above models, we mutated Lys352 or His355 in human GPRC6A, into Glu352 (mutant K352E) or Pro355 (mutant H355K), and as well created double mutated Glu352/Pro355 (mutant K352E/H355P) GPRC6A. These mutations change the positive charged Lys and His to negative charged Glu and neutral charged Pro and are thus predicted to disrupt the predicted Ocn interactions. Mutants and wild‐type hGPRC6A cDNAs were transiently transfected into HEK‐293 cells and signaling was assessed in response to Ocn treatment.

We confirmed that mutants K352E, H355P, K352E/H355P, and WT mGRPC6A proteins were equally expressed, as assessed by Western blotting using a Myc antibody, which recognized the Myc epitope located at after signal peptides (1–18 amino acids) of the WT and mutant receptors (Figure S3B).

**FIGURE 6:**
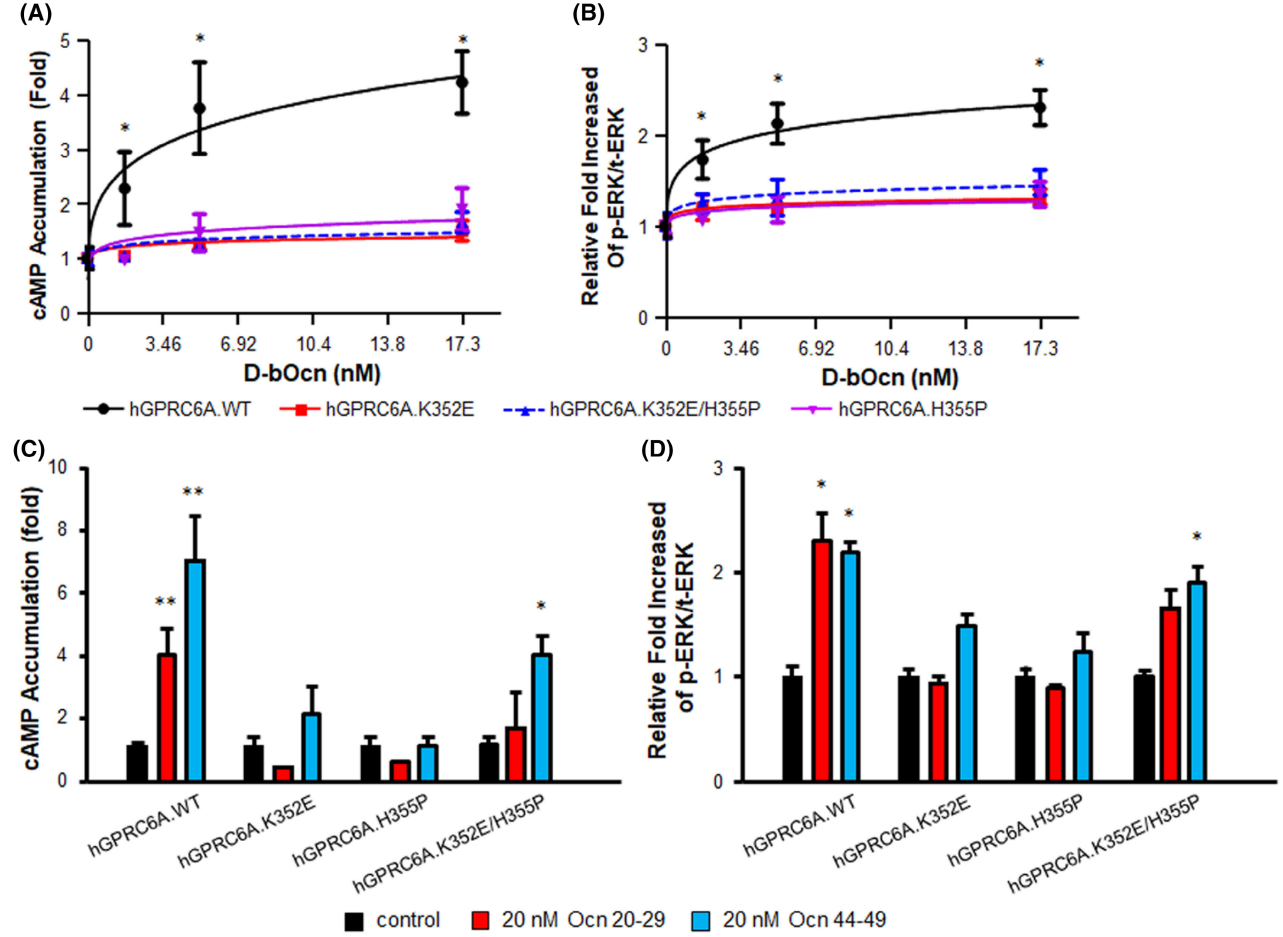
Mutagenesis of residues in predicted Ocn‐binding pocket of GPRC6A. Comparison of dose‐dependent effects of Ocn on cAMP accumulation (A) and ERK activation (B) in HEK‐293 cells transfected with the cDNA plasmids of WT, K352E, H355P, or K352E/H355P hGPRC6A. * indicate a significant difference from control (HEK‐293 cells transfected each isoforms stimulated buffer only, did not with Ocn) and stimulation groups (difference concentrations of full length Ocn) at *p* < 0.05, respectively. The Student's *t* test was performed for differences between groups. All values are expressed as means ± SEM. Comparison of Ocn small fragments, Ocn 20–29 or Ocn 44–49 at 20 nM concentration on cAMP accumulation (C) and ERK activation (D) in HEK‐293 cells transfected with the cDNA plasmids of WT, K352E, H355P, or K352E/H355P hGPRC6A. * and ** indicate a significant difference from control (HEK‐293 cells transfected each isoforms stimulated buffer only, did not with any Ocn peptides) and stimulation groups at *p* < 0.05 and *p* < 0.01, respectively. The Student's *t* test was performed for differences between groups. All values are expressed as means ± SEM.

The full‐length Ocn 1–49 peptide dose‐dependently stimulated cAMP accumulation (Figure 6A) and ERK phosphorylation (Figure 6B) in the wild‐type receptor, but the response was significantly decreased in the K352E, H355P and K352E/H355P mutant receptors (Figure 6A,B). Cells transfected with these three mutants hGPRC6A cDNAs also showed no response to Ocn 20–29 stimulation (Figure 6C,D). These results indicate that the binding sites identified by the computational modeling are accurate and that the helical region (352–363) is important for Ocn binding.

The Ocn 44–49 C‐terminal fragment, which was predicted to bind to the 7TM in our prior homology model,^11^ also had a significantly attenuated signaling response in the single mutants. Interestingly, Ocn 44–49 was able to partially activate the double mutant as assessed by cAMP accumulation and ERK phosphorylation (Figure 6C,D).

In our previous report,^11^ Ocn 44–49 was docked into a binding pocket including trans‐membrane domains and intra‐ and extra‐cellular loops, whereas the present predicted binding site for Ocn 20–29 is in the VFT of GPRC6A (Figure 5; Figure S6). Since our prior data implicated the 7TM as a putative binding site for the Ocn 44–49 peptide, we examined the effects of the Ocn C‐terminal fragment, Ocn 44–49 in activating the GPRC6A isoform with deletion of the VFT region. We found that Ocn 44–49 C‐terminal peptide activated GPRC6A signaling in all GPRC6A isoforms, including isoforms 2 and 3 (Figure S1), which had diminished responses to the Ocn 20–29 peptide (Figure 2). These results suggest that the Ocn 20–29 and the 40–49 peptides respectively bind to the VFT and TM regions of GPRC6A.

## 4 DISCUSSION

Recent cryo‐EM analysis of the Family C mGluR5^17^ and CaSR^18^, ^20^, ^21^ receptors describes the structural basis for their orthosteric ligand activation by calcium and L‐amino acids and allosteric modulating effects of peptides and small molecules. In the current study, we developed structural homology models of GPRC6A based on the above cryo‐EM results and found evidence for ligand binding sites in its VFT, similar to both the orthosteric and allosteric sites identified in mGluR5 and CaSR.

GPRC6A is activated by L‐amino acids, cations, and the Ocn peptide.^4^ Prior work predicted that the hexapeptide Ocn 44–49 C‐terminal fragment binds to the 7TM domain of GPRC6A.^11^ However, a comparison of recently available Cryo‐EM structures of mGluR5 and CaSR suggested that a regulatory binding site for peptides may exist in the VFT domains of Family C receptors. Loss of full length Ocn induced activation of GPRC6A in isoforms lacking a portion of the extracellular domain suggesting that a second binding site for Ocn may exist in the VFT. The new structural models developed for GPRC6A and complimentary experiments and peptide‐protein docking calculations reported here indeed provide additional evidence of a binding site for full‐length and/or residues 20–29 of Ocn within the GPRC6A VFT, located near the helical region (352–363). Cryo‐EM studies of GPRC6A bound to Ocn will be needed to further experimentally validate these predictions.

Reports of GPRC6A activation by the peptide Ocn are inconsistent, with some studies showing activation^2^, ^3^, ^5^, ^11^, ^35^ and other studies finding no effect.^36^ Here, we confirmed Ocn activation of GPRC6A in in vitro culture models. Moreover, the lowering of glucose and stimulation of IL‐6 and FGF‐21 at 24 h after a single injection of Ocn 20–29 in wild‐type mice but not *Gprc6a* ^*−/−*^ mice validate that these in vivo effects of this peptide are mediated through GPRC6A.

Regardless, the location of the Ocn binding site in the VFT at a site distinct from the orthosteric binding site for its natural calcium and L‐Arg ligands suggests that Ocn functions as a PAM rather than an orthosteric ligand. The fact that Ocn activation of GPRC6A also requires the presence of calcium^3^, ^5^, ^11^ in vitro is consistent with Ocn function as a PAM.^2^, ^11^ PAMs have been shown to have various effects, and in the present case we postulate that Ocn partially activates GPRC6A and stabilizes a specific conformation of the receptor similar to as previously reported for mGluR5.^17^ The requirement of orthosteric ligands for Ocn activation of GPRC6A may account for the variable ability of Ocn to activate this receptor in some studies.^36^

Our findings also suggest altered ligand specificity and downstream signaling of GPRC6A isoforms created by alternative splicing. The loss of a segment of the VFT in isoform 2 resulted in the loss of responses to L‐Arg as well as Ocn. In contrast, isoform 3 retains responsiveness to orthosteric activation but exhibits biased signaling to Ocn, as evidenced by loss of ERK but preservation of cAMP signaling. Loss of this segment may alter the conformational changes leading to opening of binding sites in the intracellular TM 6 domain that are important for G‐protein coupling. If so, different tissue distributions or ratios of the isoforms would be expected to change orthosteric ligand and PAM sensitivities as well as to alter coupling to downstream signaling pathways resulting in tissue‐specific regulation mechanisms.

Our modeling data shows that most contacts between Ocn and GPRC6A are driven by the structural part of the Ocn peptide (3 helical structure). The hexapeptide 20–29 most strongly activated GPRC6A, consistent with its location in the crucial region of the full‐length Ocn used in our docking studies to the VFT helix region. Ocn 8–19 (APVPYPDPLEPR) also activated GPRC6A (Figure S5B,C). This upstream peptide is similar to the pentadecapeptide WLGAPVPYPDPLEPR that is reported to activate GPRC6A in vitro and to improve fatty liver disease and insulin resistance after oral or intraperitoneal administration to a mouse model.^37^

We are not certain that our synthesized Ocn peptides have biological relevance. However, peptide fragments of Ocn released from bone include 1–7, 8–43, 8–19, 30–39 and 44–49 and may represent physiological ligands.^38^ Moreover, several studies^37^, ^39^, ^40^ have indicated several Ocn peptides with biological activities in serum from mouse, bovine and human, for example, metabolitin (YLGASVPSPDPLEPT). The shorter peptide 44–49 also activated GPRC6A.^11^ Previous docking and mutagenesis studies suggested that the C‐terminus of Ocn binds to the extracellular transmembrane domain of GPRC6A.^11^ This might explain the effect of the C‐terminal peptide of Ocn in activating GPRC6A in the setting of the mutations to disrupt the Ocn binding domain in the VFT helix. Overall, these data suggest there may be more than one Ocn binding site in GPRC6A.

Another interesting observation is the greater potency of the peptide Ocn 20–29 compared to the full‐length Ocn 1–49 (Figure S5A). Hypothetically this may be due to a potential disulfide bond present in the small peptide that creates a stable complementary conformation within the predicted binding site. Alternatively, competitive self‐interactions between the domain and the disordered N‐terminus of Ocn may inhibit long‐lived binding conformations between Ocn and GPRC6A. Flexibility of the N‐terminal domain that result in N‐terminal‐VFT contacts may inhibit conformational changes along the dimer interface of the VFT domains. Regardless, our data suggests that synthetic short peptides derived from Ocn might be developed to therapeutically activate GPRC6A.

We did not examine binding of other putative PAMs to GPRC6A in this study. But testosterone and triphenol compounds that activate GPRC6A are predicted to bind to the 7TM domain of GPRC6A,^13^, ^14^ analogous to the second PAM domain in the 7TM domain of mGluR5 and CaSR.^17^, ^21^ This suggests that members of the Family C GPCRs with large VFT domains have a conserved structure consisting of two allosteric binding sites, one in the VFT and the other in the 7TM domain, along with an orthosteric ligand binding domain in the VFT.

Finally, GPR158 is another proposed Ocn sensing receptor. The Cryo‐EM structure of GPR158 has recently been defined.^41^ Our VFT Ocn binding region between GPRC6A and GPR158 is not conserved and a structure superimposition of the two structures shows that the helix region (352–363) identified for GPRC6A is missing in GPR158.

Our findings are limited by the lack of Cryo‐EM structural data for GPRC6A. Cryo‐EM analysis of mGluR5 and CaSR show that each functions as homodimers, where activation leads to closure of the VFT and conformational changes the 7TMs revealing G‐protein binding sites on the cytoplasmic side necessary for signaling.^18^ For CaSR, which is most closely related to GPRC6A, receptor activation results in 7TM asymmetry in the homodimer leading to only one protomer for G‐protein coupling being stabilized by PAM binding. Our biased signaling response to Ocn in isoforms 2 and 3 suggests that there may be a structural basis for the divergent cAMP and ERK responses to Ocn that cannot be modeled with Alphafold2. To fully understand GPRC6A signaling we may need cryo‐electron microscopy structures of GPRC6A in inactive and active states bound to the orthosteric ligands, PAMS and NAMs.

## AUTHOR CONTRIBUTIONS

Rupesh Agarwal, Micholas Dean Smith and Jeremy C. Smith were responsible for the computational studies and Min Pi, Ruisong Ye, and L. Darryl Quarles were responsible for the experimental studies regarding GPRC6A. All authors contributed to the writing of the paper.

## DISCLOSURES

The authors declare no conflict of interest.

## Supporting information


Data S1.

## Data Availability

The data that support the findings of this study are available on request from the corresponding authors.
