# Supplementary material for: Osteocalcin binds to a GPRC6A Venus fly trap allosteric site to positively modulate GPRC6A signaling
Source: FASEB Bioadv. 2024 Aug 14;6(9):365–76. doi: 10.1096/fba.2024-00025 (PMC11467737; doi:10.1096/fba.2024-00025)
Supplement: Supplementary file 1 — Data S1. [file FBA2-6-365-s001.docx]

**Supporting Information for Osteocalcin binds to a GPRC6A Venus fly trap allosteric site to positively modulate GPRC6A signaling**

**Materials and methods**

**Membrane fraction isolation.**

HEK-293 transfected with human GPRC6A isoforms, mutants and vector pcDNA3.0 cDNA plasmid cells (10^5^ cells/well) (46) were cultured in 30 mm dish in DMEM supplemented with 10% fetal bovine serum and 1% penicillin/streptomycin (100 U/mL of penicillin and 100 μg/mL of streptomycin) for 48 hours. The membrane fraction was isolated by using Cell Fractionation Kit (Cell Signaling Technology) following the manufacture's protocol.

**Supporting Information**

**Figures**


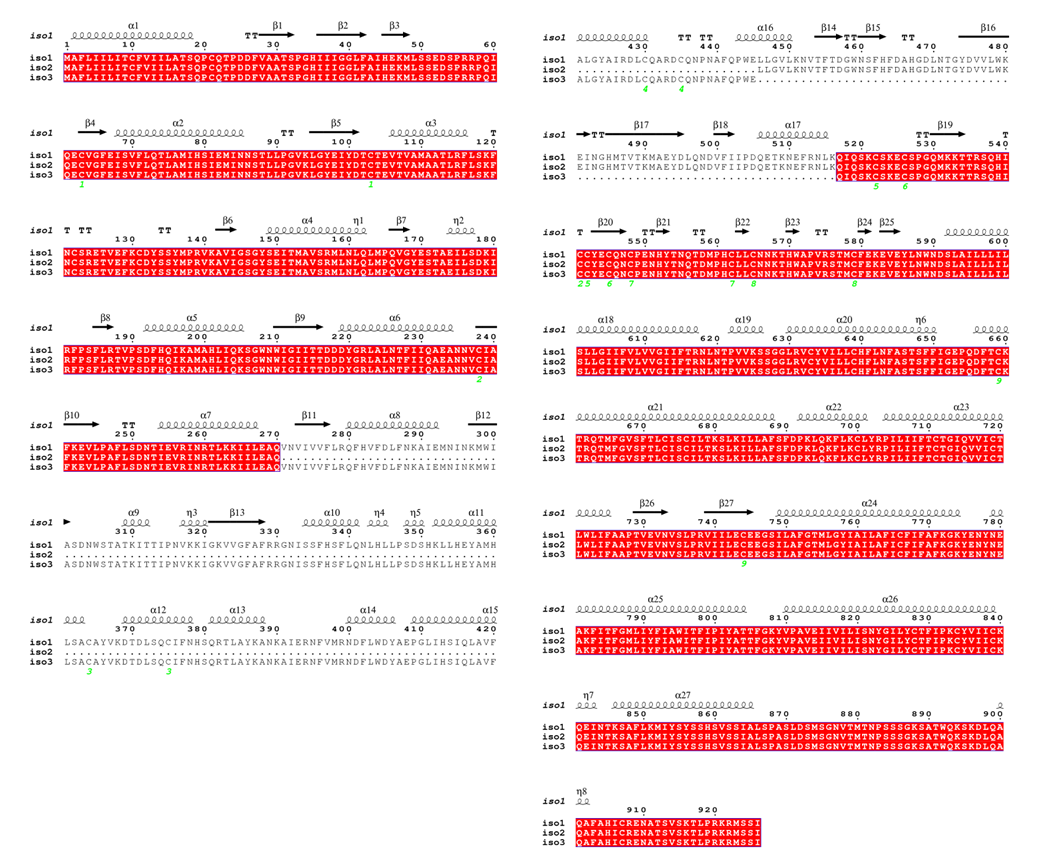


Figure S1: Sequence alignment of GPRC6A isoform 1, 2 and 3.


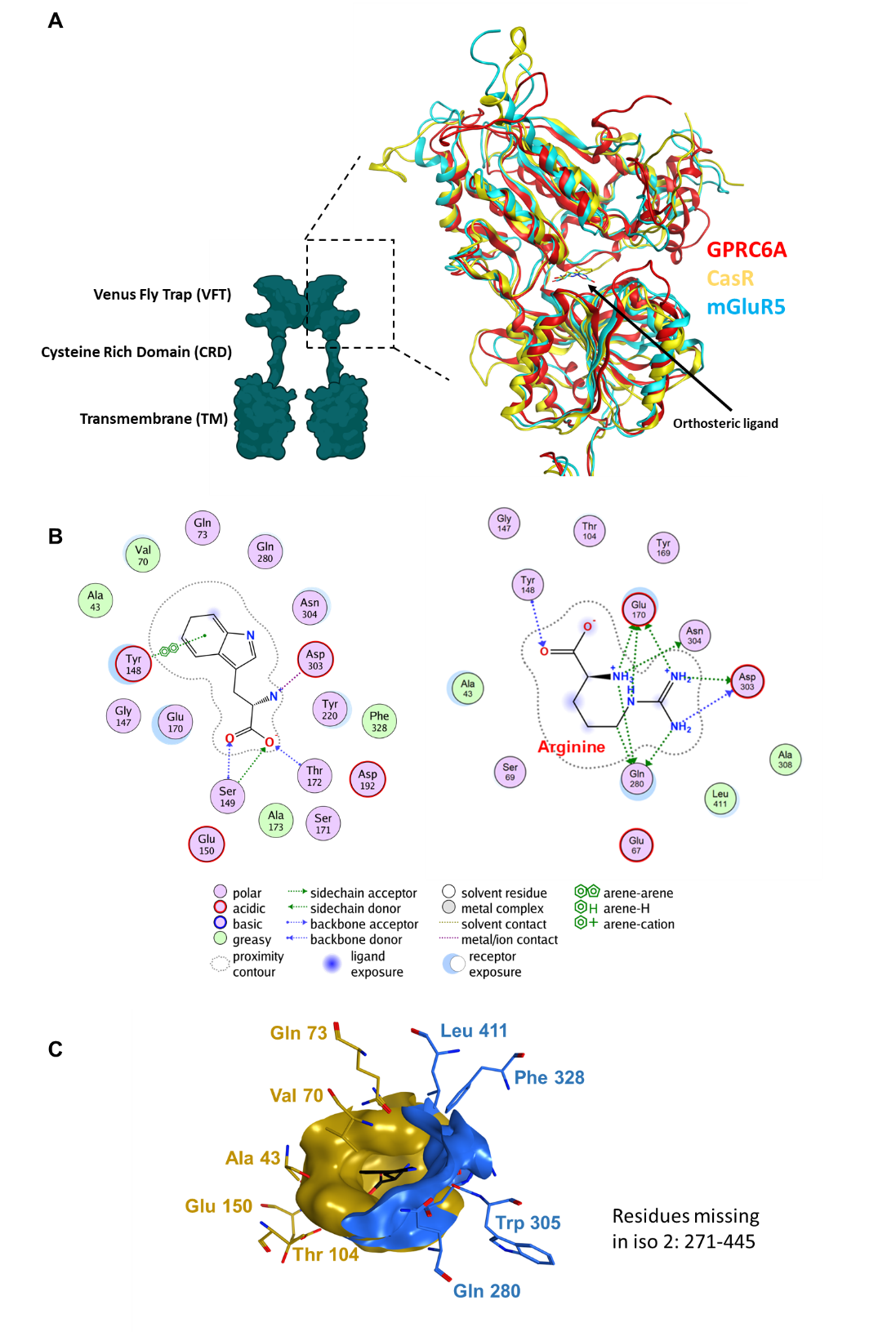


Figure S2: A. Superimposed structure of AlphaFold2 minimized model of VFT domain-GPRC6A, CaSR with L-Trp (PDB 7DTU) and mGluR5 with orthosteric analogue L-quisqualate (PDB 6N51) (in ribbon) showing orthosteric ligands. B. Interaction map of L-Trp in isoform 1 of GPRC6A showing steric pocket exists in the same place with CaSR (left panel). This model predicts that L-Arg would bind to this site in GPRC6A (right panel). C Modeling of the orthosteric ligand from CaSR in GPRC6A-isoform 1. Black: Trp (placed using CaSR:Trp as template); Blue (orthosteric residues/ orthosteric binding pocket region missing in iso2); yellow: iso1 orthosteric pocket region.


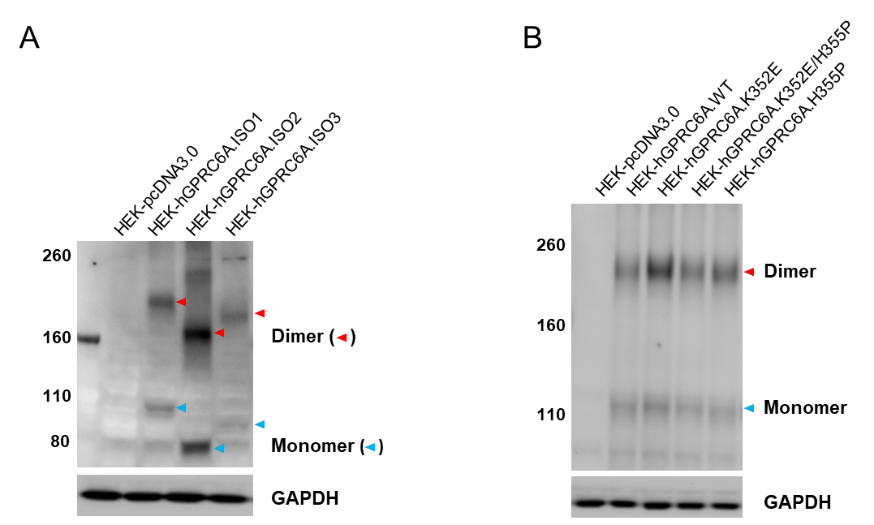


Figure S3. The protein expression of GPRC6A isoforms (A) and mutants (B).

**
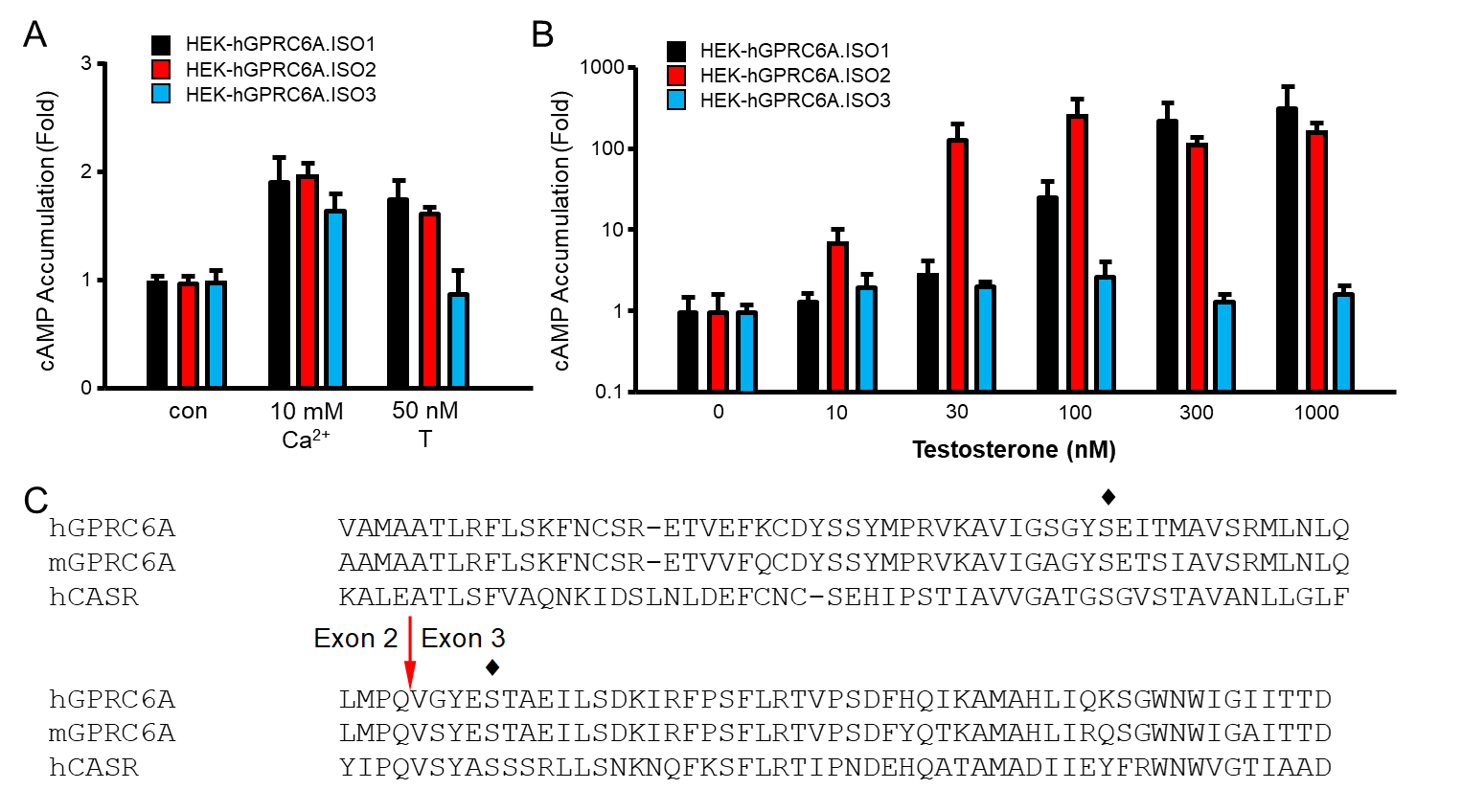
**

Figure S4. GPRC6A isoform 3 loss responsive to testosterone (T) activation. A. Comparison of effects of Ca^2+^ or T on GPRC6A isoforms mediated cAMP accumulation. B. Comparison of dose-dependent effects of T on GPRC6A isoforms mediated cAMP accumulation. HEK-293 cells were transfected with cDNA plasmids of GPRC6A isoform 1, 2 or 3 for 48 hours, after incubated in Dulbecco’s modified Eagle’s medium /F-12 containing 0.1% bovine serum albumin quiescence media for 4 hours, then exposed to Ca^2+^ or T at indicated concentrations for 40 minutes for cAMP accumulation details as described under “Methods”. * and ** indicate a significant difference from control and stimulation groups at p<0.05, and 0.01. C. Alignment of human GPRC6A, mouse GPRC6A and human CaSR showing predicted Ca^2+^ binding sites. Red arrow shows the junction of exon 2 and exon 3. ♦ indicates the expected amino acid in GPRC6A depended on CaSR.


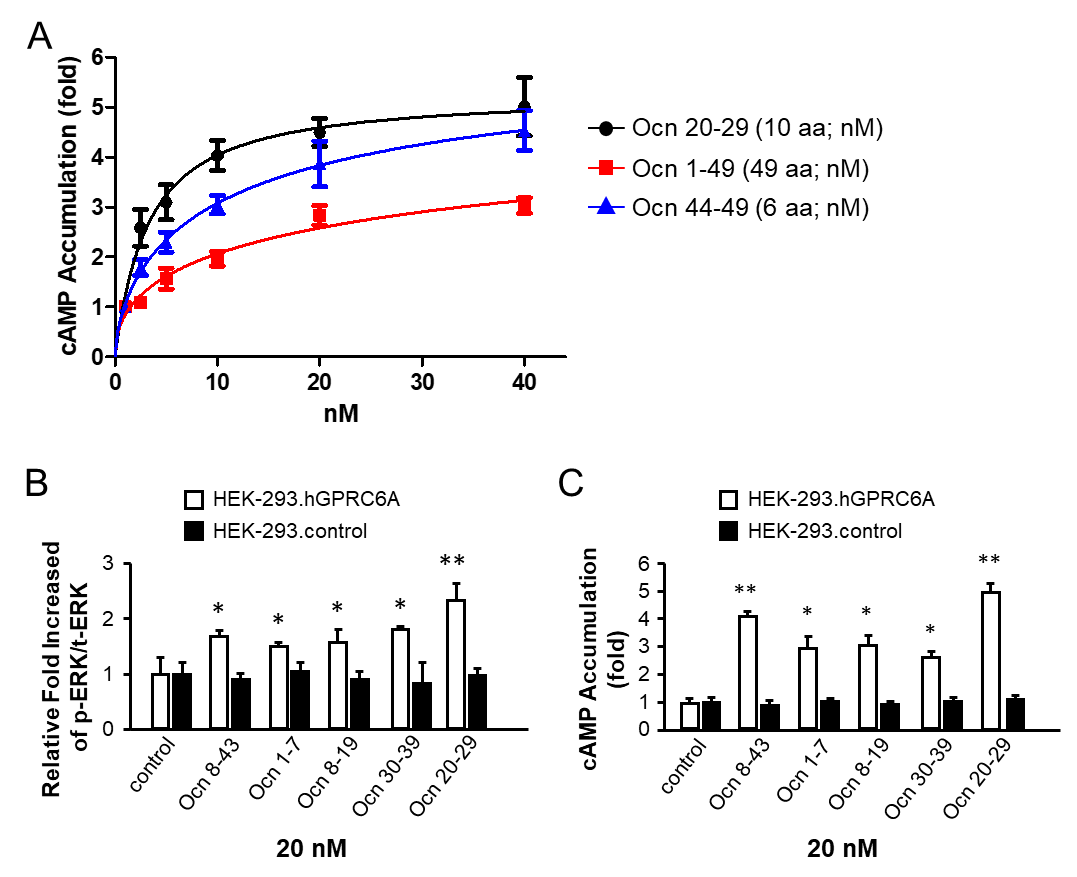


Figure S5. Human Ocn peptide fragments stimulate GPRC6A. A. Comparison of the dose response of synthesized fragments of human Ocn 1-49, Ocn 20-29 and Ocn 44-49 by cAMP accumulation in HEK-293 cells transfected with pcDNA3-hGPRC6A cDNA. Comparison of the activities of synthesized fragments of human Ocn (at 20 nM) by ERK phosphorylation (B) and cAMP accumulation (C) in HEK-293 transfected with vector pcDNA3 (black bar) and HEK-293 cells transfected with pcDNA3-hGPRC6A cDNA (white bar). * and ** indicate a significant difference from control and stimulation groups at p<0.05 and p<0.01, respectively. The ERK phosphorylation was measured 15 minutes and cAMP accumulation was measured 40 minutes for Ocn fragments at concentration as indicated at indicated stimulation in HEK-293 cells transfected with pcDNA3-hGPRC6A cDNA after 4 hours quiescence. The amino acid sequence of Ocn 8-43: AP VPYPDPLEPR REVCELNPDC DELADHIGFQ EAYR; Ocn 1-7: YLYGWLG; Ocn 8-19: AP VPYPDPLEPR; and Ocn 30-39: DELADHIGFQ.

Figure S6: A. Superimposition of crystal structures on GPRC6A model. Yellow ribbon: GPRC6A; Dark blue ribbon: region missing in iso2; green ribbon: part missing in iso3; Red ribbon: PAM Nb43 from mGluR5 (PDB 6N4Y); magenta ribbon: NAM NB2D11 from CaSR (PDB 7E6U); Cyan sphere: etelcalcetide (calcimimetic drug) (PDB 7M3G); B. AlphaFold2 minimized model of hOcn colored from N to C terminus (Blue to red) showing disulfide bond between Cys23 and Cys29.
